# MRI evaluation of vessel wall stretch in healthy and diseased aortas

**DOI:** 10.1186/1532-429X-15-S1-P249

**Published:** 2013-01-30

**Authors:** Henrik Haraldsson, Michael D Hope, Gabriel Acevedo-Bolton, Elaine E Tseng, Xiaodong Zhong, David Saloner

**Affiliations:** 1 Department of Radiology, University of California, San Francisco, San Francisco, CA, USA; 2 Department of Veteran Affairs Medical Center, San Francisco, CA, USA; 3 Department of Surgery, University of California, San Francisco, San Francisco, CA, USA; 4 MR R&D Collaborations, Siemens Healthcare, Atlanta, GA, USA

## Background

Mechanical properties of the aortic wall have been used to diagnose and characterize cardiovascular disease. Aortic stiffness correlates with aortic disease and disease progression. Mechanical regulation from strain and wall shear stress is hypothesized to influence remodeling in the cardiovascular system. Current non-invasive techniques for assessing aortic deformation are based on diameter changes, and are sensitive to through-plane motion of the aorta. Conventional methods for assessing strain, such as tagging, have limited resolution. We propose a new MRI approach that measures the regional stretch of the aortic wall itself using displacement encoding with stimulated echoes (DENSE).

## Methods

Six subjects were studied with standard CINE and DENSE imaging of the tubular portion of the ascending aorta. DENSE was performed at the point of max dilation according to the cine images. The aorta was segmented in the DENSE images and the stretch of the aortic wall was calculated.

## Results

**Figure 1:**
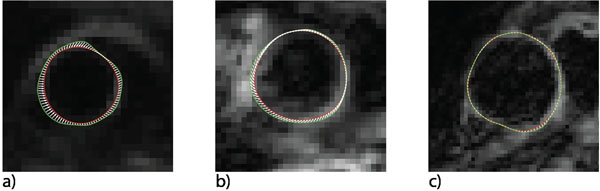
The white arrows show the relation between the systolic (green) and diastolic (red) shape of the aorta for a, a) young volunteer with stretch of 10%, b) patient with atherosclerotic aorta with stretch of 5%, and c) patient with dilated aorta with stretch of 0.4%.

Two young volunteers (26 years old) had an aortic stretch of 12%, two older patients (48 years old) with cardiovascular risk factors had stretch of 5%, and two older patients (60 years old) with dilated aortas had stretch of 1%. Figure 1 shows the circumferential stretch of 10±7% in a young volunteer (a), 5±6% in an older patient with aortic valve disease (b), and 0.4±0.4% in an older patient with a dilated aorta (c). Regional differences in stretch are noted with higher stretch seen on the right for the volunteer and the patient with valvular disease.

## Conclusions

This novel application of DENSE imaging allows for direct assessment of regional stretch in the ascending aorta. Clear differences are seen between volunteers and older patients, and within patients depending on the degree of aortic dilation present. Regional differences along the aortic lumen are evident, which cannot be imaged with other techniques. Our pilot study suggests that DENSE may play central wall in better understanding how mechanical differences in the aortic wall interrelate with disease progression.

## Funding

VA MERIT Review, Covidien/RSNA Research Scholar Grant

